# Crosstalk between Melatonin and Reactive Oxygen Species in Plant Abiotic Stress Responses: An Update

**DOI:** 10.3390/ijms23105666

**Published:** 2022-05-18

**Authors:** Quan Gu, Qingqing Xiao, Ziping Chen, Yi Han

**Affiliations:** 1 School of Biological Food and Environment, Hefei University, Hefei 230601, China; guq@hfuu.edu.cn (Q.G.); xiaoqq@hfuu.edu.cn (Q.X.); 2 State Key Laboratory of Tea Plant Biology and Utilization, Anhui Agricultural University, Hefei 230036, China; 3 National Engineering Laboratory of Crop Stress Resistance Breeding, School of Life Sciences, Anhui Agricultural University, Hefei 230036, China

**Keywords:** reactive oxygen species, nitric oxide, hydrogen sulfide, melatonin, RBOHs, signaling networks, abiotic stress

## Abstract

Melatonin acts as a multifunctional molecule that takes part in various physiological processes, especially in the protection against abiotic stresses, such as salinity, drought, heat, cold, heavy metals, etc. These stresses typically elicit reactive oxygen species (ROS) accumulation. Excessive ROS induce oxidative stress and decrease crop growth and productivity. Significant advances in melatonin initiate a complex antioxidant system that modulates ROS homeostasis in plants. Numerous evidences further reveal that melatonin often cooperates with other signaling molecules, such as ROS, nitric oxide (NO), and hydrogen sulfide (H_2_S). The interaction among melatonin, NO, H_2_S, and ROS orchestrates the responses to abiotic stresses via signaling networks, thus conferring the plant tolerance. In this review, we summarize the roles of melatonin in establishing redox homeostasis through the antioxidant system and the current progress of complex interactions among melatonin, NO, H_2_S, and ROS in higher plant responses to abiotic stresses. We further highlight the vital role of respiratory burst oxidase homologs (RBOHs) during these processes. The complicated integration that occurs between ROS and melatonin in plants is also discussed.

## 1. Introduction

In nature, many plants are constantly challenged by various abiotic environmental conditions, such as salinity, cold, heat, drought, heavy metals, and nutrient deficiencies. These stresses have important impacts on crop growth, development, and productivity [1]. Abiotic stresses affect multiple aspects of plant physiology and cause widespread damages to cellular processes [2]. Plants have evolved complex regulatory pathways to sense and respond to these stresses in a timely manner [3]. In general, abiotic stress often causes oxidative stress and cell damage through inducing excess reactive oxygen species (ROS) generation, such as superoxide anion (O_2_^•–^), hydrogen peroxide (H_2_O_2_), hydroxyl radical (·OH), and singlet oxygen (^1^O_2_) [4,5,6]. Plants have evolved sophisticated antioxidant mechanisms to modulate ROS homeostasis in response to oxidative stress [4,5]. For example, plants recruit abundant enzymatic and non-enzymatic antioxidants. Among these, superoxide dismutase (SOD), guaiacol peroxidase (POD), catalase (CAT), ascorbate peroxidase (APX), thioredoxins (TRX), peroxiredoxins (PRX), glutathione peroxidase (GPX), and glutathione reductase (GR) comprise the enzymatic antioxidant system to regulate ROS accumulation [7,8]. Non-enzymatic antioxidants, such as ascorbic acid (ASC), glutathione (GSH), tocopherol (vitamin E), and flavonoids, are also responsible for keeping ROS balanced at a basal non-toxic level [7,8]. The ascorbate-glutathione cycle (AsA-GSH cycle) is regarded as an important part of the redox hub [5].

Melatonin (*N*-acetyl-5-methoxytryptamine), known as phytomelatonin, is a modulatory agent of plant growth and stress responses, such as lateral root growth, salinity, drought, heat, heavy metals, and defense against UV-B irradiation and bacterial pathogen infection [9,10,11,12,13,14,15,16,17]. Interestingly, endogenous melatonin levels are increased rapidly by the induction of synthetic genes under the above unfavorable conditions in plants [11,12,14,15]. Melatonin acts as an antioxidant in the control of ROS levels via regulating redox enzymes (including SOD, POD, CAT, APX, GR, etc.) and metabolites (including ASC, GSH, flavonoid, anthocyanins, etc.) [11,12,17,18,19,20]. Several studies have revealed that melatonin regulates the primary and secondary metabolism via mediating the master factors of metabolic processes [10,19,20,21,22].

Moreover, any deviation from ROS balance can be thought of as a reaction to ROS signaling [8,23]. Numerous studies have revealed that ROS signaling plays a dual role, and is also beneficial to plants at specific cellular compartments during the abiotic stress process [8,23]. Multiple enzymatic systems, such as POD and plasma membrane-bound NADPH oxidase, generate ROS [24]. The respiratory burst oxidase homolog (RBOH) NADPH oxidases are the primary source of ROS production at the apoplast [25]. Superoxide is generated by NADPH oxidase and dismutated to H_2_O_2_ [26]. It has been observed that *AtrbohD* and *AtrbohF* can regulate sodium (Na) and potassium (K) transport, thus limiting Na concentrations and enhancing salinity tolerance [27,28]. *AtrbohF* also plays a vital role in mediating cadmium (Cd) uptake, chelation, and translocation [29]. Moreover, the ROS wave is required for a plant’s high light, cold, and heat tolerance as well [30,31,32].

To date, several papers have confirmed the crosstalk between melatonin and signaling molecules, such as nitric oxide (NO), hydrogen sulfide (H_2_S), and hydrogen gas (H_2_); therefore, the present paper does not elucidate it in detail [9,10,18,19,33,34,35]. Here, we systematically review the updated literature on the crosstalk between melatonin and ROS in plants upon abiotic stresses, highlight the role of RBOHs, and give perspectives for future research.

## 2. Melatonin Acts as an Antioxidant to Establish Redox Homeostasis through the Antioxidant System in Plants under Abiotic Stresses

As a master regulator, melatonin plays an important role in plant tolerance to abiotic stresses, such as salinity, cold, heat, drought, and heavy metals [9,10,11,12,13,14,15,16,17]. Our previous review systematically summarized the melatonin biosynthesis and catabolism in plants [19]. We also showed that Cd stress strongly induced melatonin accumulation via regulating the expression of genes encoding tryptophan decarboxylase (TDC), tryptamine 5-hydroxylase (T5H), *N*-acetylserotonin methyltransferase (ASMT), caffeic acid O-methyltransferase (COMT), and serotonin *N*-acetyltransferase (SNAT, also called arylakylamine *N*-acetyltransferase (AANAT)) [21]. Moreover, salinity stress up-regulated the expression *SNAT* genes and improved melatonin levels in *Arabidopsis* and *Brassica napus* [35,36]. In cucumber, cold treatment induced the expression of *TDC*, *T5H*, *SNAT*, and *COMT* genes, and thus enhanced melatonin levels [37]. Heat stress also improved the transcripts of *T5H* and *ASMT* genes and melatonin levels in tomato seedlings [38]. Similarly, drought stress up-regulated the expression of *TDC1*, *T5H*, *AANAT2*, and *ASMT1* in *Malus hupehensis* and maize plants [39]. These studies mainly found that melatonin levels were significantly induced via up-regulating the transcriptional level of melatonin biosynthesis genes in response to abiotic stress in plants. Interestingly, MzASMT9 protein levels were enhanced by salinity stress in leaves of *Malus zumi* [40]. Moreover, abiotic stress also tightly regulated the activities of melatonin biosynthesis enzymes, such as *T5H*, *TDC*, *SNAT*, and *ASMT* enzymes [41,42]. For example, high temperature elevated SNAT and ASMT activity, and increased melatonin levels in rice seedlings [41]. Salinity stress induced serotonin accumulation and *N*-acetylserotonin O-methyltransferase (HIOMT) activity in vascular bundles and the cortex, leading to melatonin accumulation in sunflower (*Helianthus annuus*) plants [42].

In general, these stresses caused endogenous melatonin accumulation, indicating that melatonin might be involved in a plant’s tolerance to abiotic stress. A series of studies found that the application of exogenous melatonin increased the level of endogenous melatonin, thereby improving plant tolerance to abiotic stress [20,21,36,43,44]. For example, the endogenous melatonin content was increased in maize by application of exogenous melatonin upon both control and Al stress conditions [43]. Moreover, this increase significantly mitigated Al-induced oxidative stress [43]. Similar results were found in the role of exogenous melatonin application in the alleviation of Cd-induced growth inhibition of mallow (*Malva parviflora*) plants [20]. Pharmacological studies also revealed that exogenous melatonin application improved resistance to salinity and drought stresses via the modulation of photosynthesis and starch/sucrose metabolism in soybean [21]. The application of melatonin partly counteracted salinity-induced seedling growth inhibition in rapeseed (*Brassica napus* L.) [36]. The role of exogenous melatonin in reducing the severity induced by heat stress in wheat seedlings was also evaluated [44].

Recently, several general genetic studies have been conducted in plants [10,11,12,35,45,46,47]. In these studies, it was demonstrated that both the *TDC*-silenced mutant and *COMT1*-silenced mutant showed a lower level of melatonin in tomato plants [10]. In Arabidopsis, we found that the *atsnat* mutant showed a low content of melatonin, and appeared hypersensitive to salinity stress in comparison with the wild-type seedlings [12,35]. It was also observed that both *atsnat-1* and *atsnat-1* showed sensitivity to high levels of light [46]. The transgenic *Arabidopsis* seedlings overexpressing alfalfa *SNAT* enhanced melatonin accumulation and exhibited more resistance to Cd stress than wild-type plants [11]. In tomato plants, overexpressing *AANAT* or *HIOMT* enhanced melatonin accumulation and improved drought tolerance [45]. Heterologous expression of *HIOMT* in apple leaves showed higher melatonin levels and improved salinity stress tolerance [46]. Nevertheless, there is still much to be learned about the post-translational modulation of melatonin biosynthesis genes and the regulation of related proteins, which should be further studied in the future.

**Figure 1:**
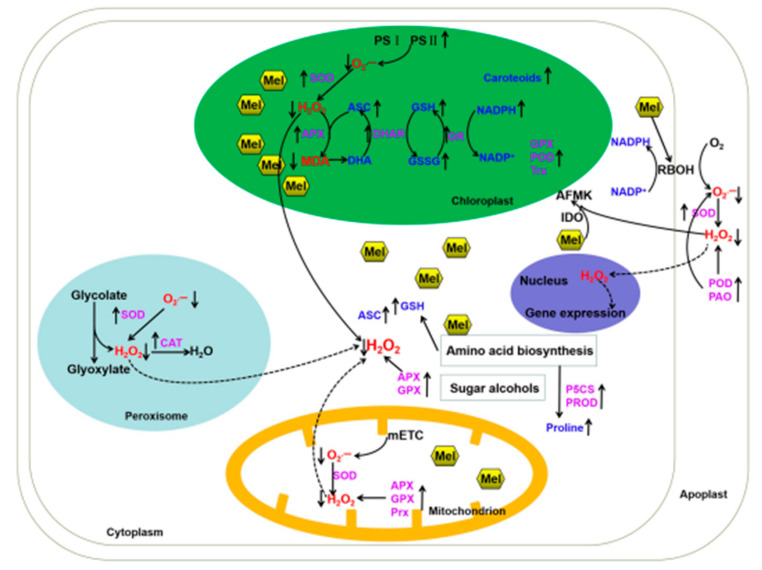
The relationship between melatonin and ROS in plant responses to abiotic stresses. Abiotic stresses, such as salinity, heat, cold, drought, and heavy metals, induce melatonin (Mel) and ROS (mainly O_2_^•–^, H_2_O_2_, and MDA) accumulation. ROS generates in excess in chloroplast, peroxisome, mitochondria, the cell membrane, and apoplast. Melatonin further regulates the activity of several antioxidant enzymes and the contents of antioxidants. Moreover, melatonin modulates the RBOH involved in H_2_O_2_ accumulation, and thereby acts as a signaling molecule to regulate gene expression in the nucleus. It is also suggested that ROS interact with melatonin to form AFMK via IDO enzyme. Melatonin regulates the amino acid biosynthesis, sugar alcohols, and carotenoids to alleviate abiotic stresses. Mel, melatonin; PS, photosystem; mETC, the electron transport chain in mitochondria; ROS, reactive oxygen species; O_2_^•–^, superoxide anion; H_2_O_2_, hydrogen peroxide; SOD, superoxide dismutase; APX, ascorbate peroxidase; POD, guaiacol peroxidase; CAT, catalase; GR, glutathione reductase; DHAR, dehydroascorbate reductase; GPX, glutathione peroxidase; Trx, thioredoxins; Prx, peroxiredoxins; P5CS, pyrroline-5-carboxylate synthetase; PROD, proline dehydrogenase; IDO, indoleamine 2,3-dioxygenase; PAO, polyamine oxidase; ASC, ascorbic acid; DHA, dehydroascorbate; GSH, reduced glutathione; GSSG, oxidized glutathione; AFMK, *N*^1^-acetyl-*N*^2^-formyl-5-methoxykynuramine; RBOH, respiratory burst oxidase.

Abiotic stresses cause endogenous ROS (mainly O_2_^•–^, H_2_O_2,_ and MDA) accumulation in plants, which generates in different various organelles including chloroplast, peroxisome, mitochondria, and the cell membrane during abiotic stresses (Figure 1) [4]. For example, O_2_^•–^ acts as the by-product of oxygen reduction by the electron transport chain (ETC) in chloroplast and mitochondria [48,49]. They also generate by photorespiration and fatty acid oxidation in peroxisome [50,51]. Then, H_2_O_2_ is produced from O_2_^•–^ by the activity of SOD or glycolate oxidases. Furthermore, NADPH oxidases, cell-wall-bound peroxidases (POX), and polyamine oxidases (PAO) result in ROS generation in the cell membrane, cell wall, and apoplast, respectively [4,52]. As toxic byproducts, ROS could cause damages to the RNA, DNA, and proteins of plants (oxidative stress situations) [8].

Melatonin acts as a potential antioxidant against abiotic stresses in plants. Afterwards, melatonin enhances the tolerance via up- or down-regulating downstream regulating elements within the physiological environments of various plants (Figure 1, Table 1). The increased melatonin decreases O_2_^•–^ and H_2_O_2_ accumulation via the enhanced antioxidant enzyme activities and antioxidant levels [9,10,18,19]. Some examples of the various roles of melatonin in the regulation of redox homeostasis in plants under abiotic stresses are illustrated in Table 1. Salinity stress is one of the serious threats to crop growth and development. Many studies indicate that melatonin enhances tolerance to salinity stress in various plant species, including *Arabidopsis*, *Brassica napus*, rice, wheat, tomato, cucumber, *Malus domestica*, *Limonium bicolor*, sunflower, and olive [12,35,36,40,42,47,53,54,55,56,57,58,59]. Within these studies, melatonin regulated ion homeostasis, especially Na^+^ and K^+^ homeostasis, thus alleviating the salinity damage. Melatonin treatment up-regulated the expression of *SOS1*, *NHX1*, and/or *AKT1*, and then maintained K^+^/Na^+^ homeostasis in *Arabidopsis* and rice [12,35,36,53]. Moreover, to reestablish the redox homeostasis, melatonin also enhanced the expression of genes encoding antioxidant enzymes (such as *APX1*, *APX2*, *CAT1*, *FSD1*, *CuZnSOD*, and *MnSOD*), and improved the activities of APX, SOD, CAT, POD, Δ1-pyrroline-5-carboxylate synthesis (P5CS), as well as the levels of antioxidants (ASC, GSH, proline, and total soluble carbohydrates) [12,35,36,40,42,47,54,55,56,57,58,59] (Table 1). Similarly, it was well established that melatonin boosted the activities of many antioxidant enzymes (including SOD, POD, APX, CAT, DHAR, GST, GR, MDHAR, and PPO) and the levels of antioxidants (including ASC, DHA, GSH, proline, flavonoid, carotenoid, and phenolic compounds), thus reducing ROS levels and improving tolerance to drought stress in plants, such as maize, tomato, citrus, soybean, *Malus*, or kiwifruit plants [14,39,60,61,62,63,64] (Table 1). Melatonin also acted as a priming agent to improve *Medicago sativa* tolerance to drought stress via the nitro-oxidative homeostasis [65]. Both cold and heat stress can induce ROS accumulation and alter the redox homeostasis. The increase in melatonin alleviated the inhibition of germination and growth of plants, such as *Arabidopsis*, rice, watermelon, *Camellia sinensis*, cucumber, tomato, soybean, *Chrysanthemum*, *Actinidia deliciosa*, etc. [15,37,38,44,66,67,68,69,70,71,72,73,74,75,76,77] (Table 1). Similar to salinity and drought stresses, cold or heat stress induced severe oxidative stress, and melatonin increased APX, SOD, CAT, POD, GPX, GR, Gly I, and Gly II activity, as well as GSH, ASC, proline, flavonoid, and proline contents. Furthermore, melatonin treatment positively modulated *ZAT10* and *ZAT12*, which encode transcriptional regulators of ROS-related antioxidant genes [66]. Several studies suggested that heat shock proteins (HSPs) were also involved in melatonin-regulated heat tolerance in plants, such as *Arabidopsis*, tomato, and kiwifruit [74,76,77]. In addition, heavy metal pollutants were shown to induce serious stress and toxicity in plants. Melatonin protects plants upon heavy metal stress, such as Cd, aluminum (Al), lead (Pb), mercury (Hg), copper (Cu), vanadium (V), and arsenic (As) [11,19,20,43,78,79,80] (Table 1). In these studies, melatonin improved Cd-triggered redox imbalance through changes in Cu/ZnSOD genes, which are regulated by miR398a and miR398b [11]. Seeds of red cabbage with melatonin pretreatment conferred Cu tolerance by blocking the membrane peroxidation and DNA damages [78]. Treatment of exogenous melatonin or improvement of endogenous melatonin by overexpressing the melatonin synthetic-related genes stimulated the activities of antioxidant enzymes (including APX, SOD, CAT, POD, GPX, GR, and PAL) and increased antioxidant levels (including DHA, GSH, proline, flavonoid, and anthocyanins), thus inhibiting ROS production in plants, such as tomato, *Nicotiana tabacum* L., rice, maize, wheat, *Azolla imbricata*, and watermelon seedlings under the stress of heavy metals [11,19,20,43,78,79,80] (Table 1). Melatonin also improved the efficiency of PSII and regulated amino acids, sugar alcohols, and carotenoids metabolism to enhance plant tolerance to abiotic stress (Figure 1).

## 3. Plant Abiotic Stress Tolerance Is Mediated by the Crosstalk between Melatonin and Signal Molecules (NO, H_2_S, and ROS)

Melatonin was shown to be a crucial regulator occupying extensive roles in many physiological and biochemical processes throughout plant life, especially plant responses to abiotic stress [9,10,19]. Furthermore, melatonin was shown to function with other signal molecules in order to manipulate environmental damages, such as NO, H_2_S, ROS, and H_2_ [9,10,18,19,33,34,35]. The latest reviews systematically revealed the inter-relationship between melatonin and gasotransmitters (including NO, CO, H_2_S, CH_4_, and H_2_) in resistance to plant abiotic stress [10,81,82,83]. For example, melatonin altered the endogenous NO accumulation, and reduced reactive nitrogen species (RNS) (ONOO–, and peroxynitrous acid), which was generated by stress [81,82]. Nevertheless, melatonin regulated the expression of the nitric oxide synthase (*NOS*) gene, and triggered endogenous NO accumulation [84]. Ample evidence manifested that NO acted as the downstream signal of melatonin to regulate plant tolerance to salinity, drought, heat, cold, Cd, and Al stresses [33,34,36,85,86,87,88]. Zhao et al. found that melatonin enhanced rapeseed seedling tolerance via NO signaling against salinity stress, and similar results were obtained for sunflower seedlings as well [36,85]. Melatonin down-regulated the NO accumulation, thus promoting soybean tolerance to drought stress [86]. Moreover, positive and antagonistic interactions between melatonin and NO might exist in plant responses to stress caused by heavy metals [19,87,88].

It was also shown that H_2_S plays a vital role in enhancing plant tolerance to abiotic stress and alleviating its detrimental effects [89,90,91,92]. Melatonin increased the activities of the H_2_S-produced enzymes (*D*-cysteine desulfhydrase (DCD), *L*-cysteine desulfhydrase (LCD)), thus improving H_2_S accumulation [93,94,95]. Further, application of hypotaurine (HT, H_2_S scavenger) reversed the contribution of melatonin in alleviation of the salinity and heat damages by reestablishing the redox homeostasis in tomato, cucumber, and wheat seedlings [44,94,95]. Kaya et al. found that the interactive effect of NO and H_2_S improved the wheat’s resistance to Cd stress via enhancing the antioxidative defense system and reducing the damage induced by oxidative stress [96]. Moreover, the H_2_S and NO jointly were involved in melatonin-regulated salinity tolerance in cucumbers [95]. They were also involved in melatonin-mediated resistance to iron deficiency and salinity stress in pepper seedlings [97]. Until now, the interactions among melatonin, NO, and H_2_S in plant responses to abiotic stress were not largely explored.

In recent years, apart from NO and H_2_S, great efforts were made in studies conducted on ROS-directed plant abiotic stress responses [4,5,6,7,8]. In this review, Section 2 shows that ROS are inevitably produced by adverse environmental conditions, and thereby significantly cause damages to the structural and functional integrity of the whole plant seedling. More importantly, ROS instantly produced in chloroplast, peroxisome, mitochondria, and cell membrane organelles often modulate signaling pathways when maintained at a moderate concentration [4,5,6,7,8]. Recent studies have further shed new light on the role of ROS in melatonin-regulated tolerance to abiotic stresses in plants. In early responses to cold stress, melatonin was found to stimulate H_2_O_2_ accumulation in watermelon [98]. Chen et al. found that endogenous melatonin rapidly induced ROS accumulation under short-term salinity treatment in *Arabidopsis* [12]. Then, ROS triggered SOS-mediated Na^+^ efflux and intensified the increased antioxidant defense [12]. Similarly, melatonin triggered an ROS burst that enhanced the expression of K^+^ uptake transporters to enable K^+^ retention under salinity stress in rice [53]. H_2_O_2_ scavengers negated the effects of melatonin-mitigated abiotic stress, such as drought, heat, and cold stress in tomato plants [99]. Collectively, these studies preliminarily revealed that ROS signaling acts downstream of melatonin in alleviation of abiotic stress in plants.

Several articles have also shown the mechanisms underlying the complexity of ROS with NO and/or H_2_S signaling in plant tolerance against abiotic stress [2,3,4,89,90,92]. Zeng et al. reviewed the crosstalk among melatonin, NO, and ROS in plant tolerance to bacterial, fungal, and viral diseases [100]. This phenomenon was also shown to promote fruit ripening [9]. However, more advances should be made to provide new insights on the understanding of the crosstalk among melatonin, NO, H_2_S, and ROS in plant abiotic tolerance using genetic, pharmacological, genomic, and proteomic approaches.

## 4. The Roles of RBOH-Involved ROS Signaling in Melatonin-Modulated Plant Processes

ROS produced in several organs (the cell membrane, chloroplast, peroxisome, mitochondria, and apoplast) are implicated in signaling pathways (Figure 1). Respiratory burst oxidase homolog (RBOH) proteins are the NADPH oxidases localized on the plasma membrane [101]. They are the key proteins associated with the signal transduction event [101]. There is a C-terminal FAD/NADP(H)-binding domain, a N-terminal regulatory domain, six transmembrane domains, and several potential phosphorylation sites in *RBOHs* [24,25]. The NADPH oxidases are modulated by phosphorylation and/or binding of calcium ions at the cytosol, and then produce O_2_^•−^ at the apoplast (Figure 1). O_2_^•−^ is converted into H_2_O_2_ via the action of SOD, or spontaneously [8,101]. Afterwards, H_2_O_2_ could enter various types of cells and trigger different signaling responses. There are ten or eight *RBOH* genes encoding NADPH oxidase in Arabidopsis or tomato, respectively [24,25]. In recent years, many studies have shed light on ROS-directed plant growth and stress responses in *Arabidopsis* and other crops. In this review, we emphasize the roles of RBOH-involved ROS signaling in melatonin-mediated plant abiotic stress tolerance.

Recently, the roles of Rbohs were analyzed in *Arabidopsis*, tomato, tobacco, rice, cucumber, alfalfa, etc. [12,53,102,103,104,105,106]. Most of these genes played important roles in resistance to salinity, high/low temperature, heavy metals, and biological stress [12,53,102,103,104,105,106]. It was reported that *AtRbohC* regulated the stability of *SOS1* mRNA to improve salinity tolerance [102]. *AtrbohC*, *AtrbohD*, and *AtrbohF* also inhibited calcium (Ca), zinc (Zn), and iron (Fe) translocation [29]. *SlRbohB*-involved ROS signaling was required for tolerance to drought stress in tomatoes [103]. Meanwhile, *RBOH1*-produced H_2_O_2_ induced the expression of downstream genes to enhance tomato’s resistance to cold, salinity, and salinity–alkalinity stresses [104,105,106]. In tobacco, the *NtRbohE*-derived ROS signaling pathway improved salinity tolerance [107]. Moreover, RBOH-mediated ROS production was involved in lateral root growth, and AtRbohC promoted root hair budding in Arabidopis [108,109]. *AtrbohF*-mediated H_2_O_2_ signaling acted as a key mediator in stomatal closure in guard cells [110]. Hence, *RBOHs*-involved ROS signaling plays a vital role in plant growth and abiotic stress tolerance.

Much more is now known about how *RBOH* genes are regulated in response to abiotic stresses. Recent evidence suggests that ROS function as signaling molecules in relation to hormone responses, and the mechanistic bases are complicated. Clearly, melatonin has complex crosstalks with signaling molecules and other phytohormones [19,111]. In our studies, melatonin-triggered lateral root formation was H_2_O_2_-dependent in alfalfa [13]. Further, melatonin induced PAO and *RBOH*-derived ROS accumulation to facilitate the lateral root development in tomatoes [112,113]. The roles of *PuRBOHF*-dependent H_2_O_2_ were also essential for melatonin-induced anthocyanin accumulation in red pear fruit [114,115]. Moreover, a *RbohF*-dependent ROS burst was required for melatonin-triggered salinity tolerance in *Arabidopsis* and rice [12,53]. So far, the ways that most *RBOHs* function in melatonin-regulated processes in response to abiotic stress are still elusive, and it should be clarified in future studies.

## 5. How Melatonin Directs with the RBOH-Regulated ROS Signaling in Plant Tolerance to Abiotic Stress

**Figure 2:**
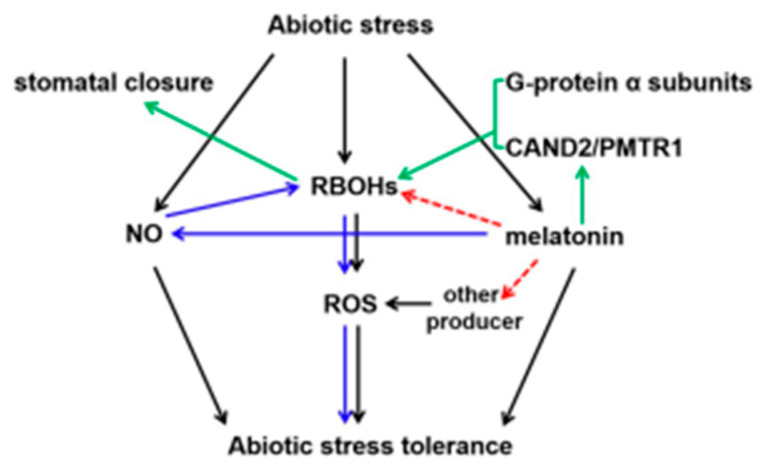
Probable integrative model of ROS with melatonin regulator in plant responses to abiotic stress. Increasing evidence shows that melatonin induces NO generation and enhances RBOH activity through denitrosylation, thereby activating ROS signaling in tomatoes (blue arrow). Interaction between CAND2/PMTR1 (the melatonin receptor) and G-protein α subunits activates *RBOHs,* resulting in stomatal closure (green arrow). The direct relationship between the melatonin and RBOHs in plant tolerance to abiotic stress is still largely unknown (red arrow, yet largely unknown). NO, nitric oxide; ROS, reactive oxygen species; RBOHs, respiratory burst oxidase homologs.

Several studies take the important stance that the transmembrane receptor of melatonin (PMTR1/CAND2) is found in *Arabidopsis*, tobacco, alfalfa, and maize [116,117,118,119]. It is also located in the plasma membrane, and can interact with G-protein α subunits, thereby activating *RBOHs* to promote stomatal closure in Arabidopsis (Figure 2) [116,119]. Melatonin inhibits endogenous NO accumulation and reduces the S-nitrosylation of RBOH to activate the ROS signaling pathway [99]. ROS signaling induces the expression of defensive genes to enhance plant tolerance to oxidative stress [120,121]. However, whether or how the interaction between PMTR1/CAND2 and G-protein α subunits directly regulates RBOHs in plant response to abiotic stress remains to be deciphered.

Recent data imply that melatonin connects with the multiple elements, including the hormone and signaling molecules. Moreover, it was found that H_2_S modulates the post-translational modification of protein cysteine residues in plants [122,123,124]. Our previous review further suggested that ROS interacted with H_2_S by regulating transcriptional or post-translational modifications in response to oxidative stress [125]. Therefore, interaction of melatonin with ROS and H_2_S in regulating abiotic stress also has significant importance and remains to be identified in future.

## 6. Conclusions and Perspectives

Much more is now known about the regulatory mechanism of melatonin-mediated tolerance to abiotic stresses, especially the cooperation between melatonin and ROS, NO, and/or H_2_S. To further promote this research in plants, our review summarizes the ROS-involved regulatory roles and mechanisms of melatonin-mediated abiotic stress resistance. Melatonin confers oxidative stress tolerance mainly through the reestablishment of redox homeostasis. Moreover, ROS act as signaling molecules that regulate melatonin-modulated protective effects. In particular, the vital role of RBOHs during these processes was shown. However, there are still many questions that should be characterized to understand the signal transduction pathway of melatonin in plants in response to abiotic stress. For example, it is necessary to focus more attention on the signaling role of ROS produced by photosystem II (PSII) and photorespiration in melatonin-alleviated abiotic stress in future studies.

As melatonin is an important regulatory element of phytohormones, it collaborates with multiple elements (such as the discovered signaling molecules NO, H_2_S, and ROS) and hormones (such as auxin, ethylene, salicylic acid, gibberellin, and abscisic acid signaling). Importantly, most studies do not provide solid in vivo evidence. Future studies using related mutants produced by gene editing technology and plastid transformation technology [126,127] should aim to illustrate how melatonin functions with these signaling molecules in plants under stressful situations.

## Figures and Tables

**Table 1:** Some examples of the roles of melatonin in regulating redox homeostasis in plants under abiotic stresses.

Abiotic Stressors	Impact on Oxidative Stress Markers and Antioxidative Defense Systems (Enzymes and Related Genes)	Plant Species	References
Salinity stress	H_2_O_2_, O_2_^•–^, MDA, ·OH, and EL; APX, SOD, CAT, POD, Δ1-pyrroline-5-carboxylate synthetase, ASC, GSH, proline, and total soluble carbohydrates; *APX1*, *APX2*, *CAT1*, *FSD1*, *CuZnSOD*, and *MnSOD*	*Arabidopsis*, *Brassica napus*, *Malus domestica*, olive, tomato, wheat, cucumber, rice, *Limonium bicolor*	[12,35,36,40,42,47,53,54,55,56,57,58,59]
Drought stress	H_2_O_2_, MDA, O_2_^•–^, and EL; APX, SOD, CAT, POD, DHAR, GST, GR, MDHAR, PPO, ASC, DHA, GSH, proline, flavonoid, carotenoid, and phenolic compounds; *Cu/ZnSOD*, *Fe/MnSOD*, *APX*, *CAT*, *GR*, *POD*, *GST*, *DHAR*, and *MDHAR*	maize, tomato, citrus, soybean, kiwifruit, Malus	[14,39,60,61,62,63,64,65]
Cold stress	H_2_O_2_, O_2_^•–^, MDA, and EL; APX, SOD, CAT, POD, GR, GSH, ASC, proline, polyamine; *APX*, *CAT*, *SOD*, *GR*, *ZAT10*, and *ZAT12*	*Arabidopsis*, watermelon, *Camellia sinensis*, rice, cucumber, tomato	[15,37,66,67,68,69,70]
Heat stress	H_2_O_2_, O_2_^•–^, MDA, and EL; APX, SOD, CAT, POD, GPX, GR, Gly I, Gly II, GSH, ASC, proline, flavonoid, proline, polyamine, and carotenoid; *APX*, *CAT*, *SOD*, *POD*, *HsfA2*, and *Hsp90*	rice, soybean, maize, *Chrysanthemum*, *Actinidia deliciosa*	[38,44,71,72,73,74,75,76,77]
Heavy metals stress	H_2_O_2_, O_2_^•–^, MDA, and EL; APX, SOD, CAT, POD, GPX, GR, PAL, ASC, DHA, GSH, proline, flavonoid, anthocyanins *APX*, *CAT*, *POD*, *SOD*, *GR*, *GSH1*, *PCS*	*Tomato,Nicotiana tabacum* L., *Brassica napus* L., rice, maize, wheat, alfalfa, Azolla imbricata, watermelon	[11,19,20,43,78,79,80]

H_2_O_2_, hydrogen peroxide; O_2_^•–^, superoxide anion; MDA, malondialdehyde; ·OH, hydroxyl radical; EL, electrolytic leakage; APX, ascorbate peroxidase; SOD, superoxide dismutase; CAT, catalase; POD, guaiacol peroxidase; DHAR, dehydroascorbate reductase; GST, glutathione S-transferase; GR, glutathione reductase; MDHAR, monodehydroascorbate reductase, PPO, polyphenol oxidase; ACS, ascorbate; GSH, reduced glutathione; DHA, dehydroascorbate; GPX, glutathione peroxidase; ZAT, ROS-related responsive elements; Gly, glyoxalase; HsfA, heat-shock factor; HSP, heat-shock protein; PAL, phenylalanine ammonia-lyase.

## Data Availability

Not applicable.
